# Necroptosis and neutrophil-associated disorders

**DOI:** 10.1038/s41419-017-0058-8

**Published:** 2018-01-25

**Authors:** Xiaoliang Wang, Shida Yousefi, Hans-Uwe Simon

**Affiliations:** Institute of Pharmacology, University of Bern, Bern, CH-3010 Switzerland

## Abstract

Necroptosis is a form of regulated necrosis and is dependent on a signaling pathway involving receptor interacting protein kinase-3 (RIPK3) and mixed lineage kinase domain-like protein (MLKL). Necroptosis is considered to have important functions in inflammation and, based on studies with animal disease models, is believed likely to be involved in the pathogenesis of many human inflammatory diseases. In neutrophils, necroptosis has recently been reported to be triggered by tumor necrosis factor (TNF) stimulation, ligation of adhesion receptors, exposure to monosodium urate (MSU) crystals, or phagocytosis of *Staphylococcus aureus* (*S. aureus*). Because neutrophils are involved in many kinds of tissue inflammation and disease, neutrophil necroptosis probably plays a vital role in such processes. Dissecting the signaling pathway of neutrophil necroptotic death may help to identify novel drug targets for inflammatory or autoimmune diseases. In this review, we discuss different mechanisms which regulate neutrophil necroptosis and are thus potentially important in neutrophil-associated disorders.

## Facts

- Necroptosis is one type of regulated necrosis and is dependent on RIPK3 and MLKL activities, showing morphologic features similar to necrosis.
- Death receptors, Toll-like receptors (TLRs), the IFN-α receptor (IFNAR), adhesion receptors, and DNA-dependent activator of IFN (DAI) regulatory factors have been shown to trigger RIPK3-MLKL-dependent necroptosis.
- Neutrophil necroptosis can be induced by TNF stimulation, ligation of adhesion receptors, exposure to monosodium urate (MSU) crystals, or phagocytosis of *S. aureus*.
- Reactive oxygen species (ROS) are important contributors to neutrophil necroptosis induced by ligation of adhesion receptors or MSU stimulation.
- Human neutrophils migrating to inflammatory sites can activate the RIPK3-MLKL pathway as seen in neutrophilic diseases such as cutaneous vasculitis, ulcerative colitis, and psoriasis.

## Open questions

- Is necroptosis involved in the pathogenesis of human inflammatory diseases?
- How does neutrophil necroptosis impact on inflammatory and autoimmune diseases?
- How can one distinguish between neutrophil extracellular trap (NET) formation and necroptosis-related, passive release of chromatin?
- How is RIPK3 activated by adhesion receptors in neutrophil necroptosis?
- How is p38 MAPK activated by the RIPK3-MLKL complex?
- Are ROS initiators or executors in neutrophil necroptosis?
- How can XIAP restrict the switch to TNF-induced necroptosis in mouse neutrophils? What is the role of XIAP in human neutrophils?
- What is the executor of RIPK3-dependent regulated necrosis induced in human neutrophils by the phagocytosis of *S. aureus*?
- Why do mouse neutrophils lacking MLKL clear *S. aureus* at the site of infection only poorly?

## Introduction

**Fig. 1:**
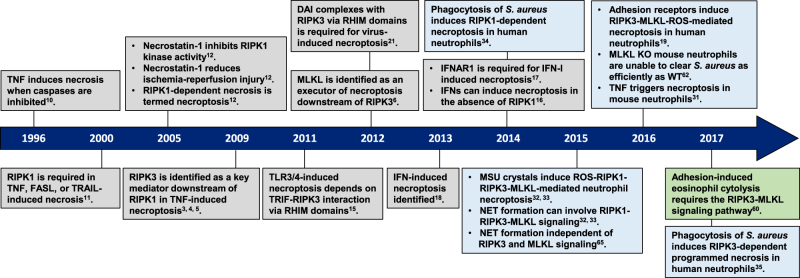
Timeline for the discovery of key proteins in the molecular pathways of necroptosis Necroptosis (Grey box); neutrophil necroptosis (Blue box); eosinophil necroptosis (Green box). *TNF* tumor necrosis factor, *FASL* FAS ligand, *TRAIL* TNF-related apoptosis-inducing ligand, *RIPK1* receptor interacting protein kinase-1, *RIPK3* receptor interacting protein kinase-3, *DAI* DNA-dependent activator of IFN regulatory factors, *RHIM* RIP homotypic interaction motif, *MLKL* mixed lineage kinase domain-like protein, *TLR* Toll-like receptor, *TRIF* Toll/IL-1 receptor domain-containing adaptor protein-inducing interferon-β, *S. aureus* *Staphylococcus aureus*, *IFNs* interferons, *IFN-I* type I interferons *IFNAR1* IFN-α receptor type I, *MSU* monosodium urate, *PMA* phorbol 12-myristate 13-acetate, *ROS* reactive oxygen species, *NET* neutrophil extracellular trap, *KO* knockout, *WT* wild-type

**Fig. 2:**
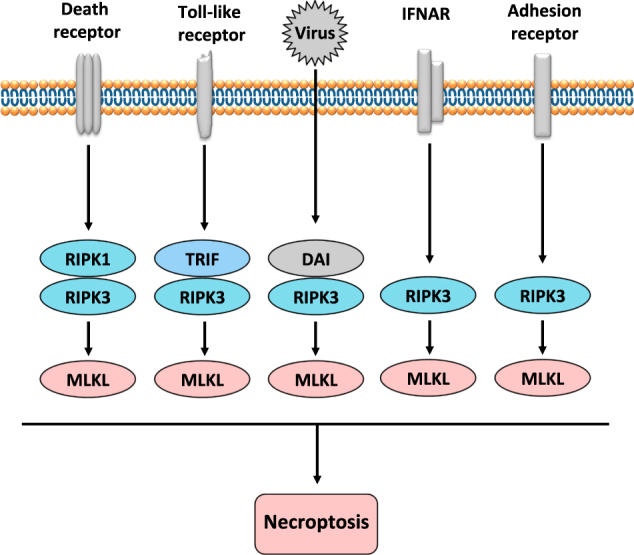
RIPK3-MLKL-mediated necroptosis induced by receptors or DAI Death receptors can trigger a RIPK1-RIPK3-MLKL-mediated necroptosis and TLRs are able to induce a TRIF-RIPK3-MLKL-mediated necroptosis. Viruses, too, can trigger a DAI-RIPK3-MLKL-mediated necroptosis as can adhesion receptors or IFN-I signaling (IFNAR), inducing a RIPK3-MLKL-mediated necroptosis. RIPK3 can be engaged by RIPK1, TRIF, or DAI through their respective RHIM domains and, furthermore, MLKL can be recruited and phosphorylated by RIPK3 to trigger necroptosis

Apoptosis was long thought to be the only form of programmed cell death during homeostasis, development and disease. The key regulators of apoptotic cell death are caspases and the characteristic morphological hallmarks, cell shrinkage, nuclear condensation, cell membrane blebbing, and formation of apoptotic bodies. In contrast, necrosis was long considered to be an unregulated and uncontrollable accidental cell death, for which the characteristic morphologic changes are cell swelling, plasma membrane rupture, and release of intracellular contents^1,2^. However, recent evidence has shown that necrosis can also occur in a regulated manner called necroptosis, now recognized as one type of regulated necrosis, dependent on RIPK3^3–5^ and MLKL^6–9^ activities and exhibiting morphologic features similar to necrosis. This necrosis-like regulated cell death can be triggered when caspases are inhibited, as was already demonstrated in 1996^10^. Since 2000, the important role of RIPK1 and of its kinase inhibitor, necrostatin-1, in this caspase-independent necrosis have been recognized^11–13^. The subsequent discovery of RIPK3 as the key protein in necroptosis^3–5^ and the identification of the pseudokinase MLKL as an effector protein downstream of RIPK3^6^ were crucial for understanding the necroptosis pathway. (Fig. 1) illustrates the history of progress in defining the necroptosis pathway. Death receptors^1,2^, Toll-like receptors (TLRs)^14–16^, IFN-α receptor (IFNAR)^16–18^, adhesion receptors^19,20^ and DNA-dependent activator of IFN (DAI) regulatory factors^21,22^, have all been shown to be involved in RIPK3-MLKL-dependent necroptosis (Fig. 2).

In contrast to apoptosis, which can limit inflammation, necroptotic cells can release massive amounts of damage-associated molecular patterns (DAMPs) from disintegrating membranes, depending on the cellular environment; thus, necroptosis is generally considered to be a contributor to inflammation^1,23^. On the other hand, necroptosis may also reduce the production of pro-inflammatory cytokines by shortening the cell lifespan, decreasing the overall inflammatory response induced by tumor necrosis factor (TNF) or lipopolysaccharide (LPS), thus suppressing the host immune response and benefiting intracellular pathogens^24^. Moreover, necroptosis is also considered to be involved in normal development^2,25^.

Neutrophils are important effector cells in innate immunity, being the most abundant population of leukocytes in the circulation^26^. They are produced in the bone marrow, where they mature before being released into the bloodstream. Neutrophils are essential for the innate immune system and rapidly migrate to inflamed tissues in response to pathogens or sterile harmful stimuli^27,28^. At the inflammatory sites, neutrophils can fight infection with an array of strategies, including phagocytosis, generation of reactive oxygen species (ROS), degranulation, and release of neutrophil extracellular traps (NETs)^29^. These functions are responsible for the vital contribution of neutrophils in fighting pathogens and in preventing spread of infection; however, when they overreact, they also damage host tissues^27^.

**Fig. 3:**
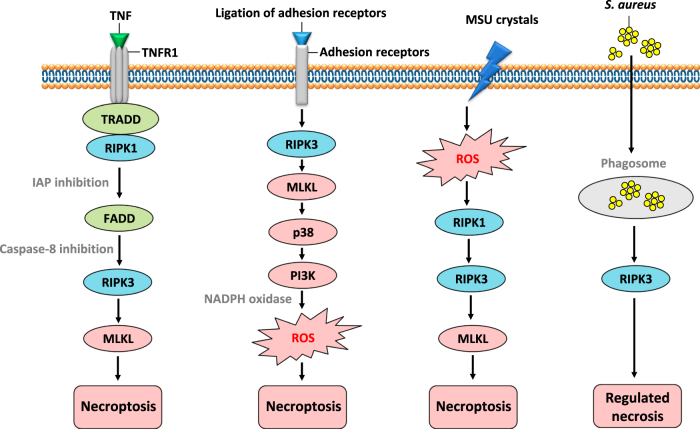
The signaling pathway of neutrophil necroptosis Neutrophil necroptosis triggered by various stimuli, including TNF, adhesion receptors, MSU crystals or phagocytosis of *S. aureus*. TNF-induced mouse neutrophil necroptosis is dependent on a RIPK1-RIPK3-MLKL signaling pathway. Ligation of adhesion receptors, including CD44, CD11b, CD18, and CD15, can induce a human neutrophil necroptosis involving the RIPK3 – MLKL - p38 MAPK - PI3K axis, in which all of these molecular components are prerequisite for generation of ROS induced by NADPH oxidase, and subsequent necroptosis. However, how the RIPK3-MLKL complex activates p38 MAPK requires further investigation and confirmation in other cell types. MSU crystal-induced neutrophil necroptosis is triggered by ROS production which further activates RIPK1-RIPK3-MLKL and subsequent cell death. The mechanism of ROS production by MSU exposure remains to be investigated. Phagocytosis of *S. aureus* can also trigger RIPK3-dependent regulated necrosis in human neutrophils

Apoptosis is the most common physiological and pathological cell death in neutrophils^28^. In the resolution of inflammation, neutrophils accumulated locally rapidly undergo apoptosis and are removed by other phagocytic cells. Therefore, neutrophil apoptosis is considered to limit tissue damage by preventing the release of histotoxic contents from dying cells^28^. In contrast, neutrophil necrosis is considered highly detrimental to the resolution of inflammation owing to the release of toxic contents and potential escape of pathogens into the surroundings^30^. Recently, several groups have reported that necroptosis can also occur in neutrophils. For instance, TNF receptor 1 (TNFR1) stimulation^31^, ligation of adhesion receptors^19,20^, activation by MSU crystals^32,33^, or phagocytosis of *S. aureus*^34,35^ can all trigger neutrophil necroptosis (Fig. 3). Neutrophils are involved in many kinds of tissue inflammation and disease;^36^ unlike apoptosis, necroptotic death of neutrophils may induce tissue injury and inflammation similar to necrosis and is thus likely to be important for the pathogenesis of infectious or autoimmune diseases. In this article, we discuss the different mechanisms regulating neutrophil necroptosis and their potential for causing neutrophil-associated disorders.

## The signaling pathway of TNF-induced necroptosis

**Fig. 4:**
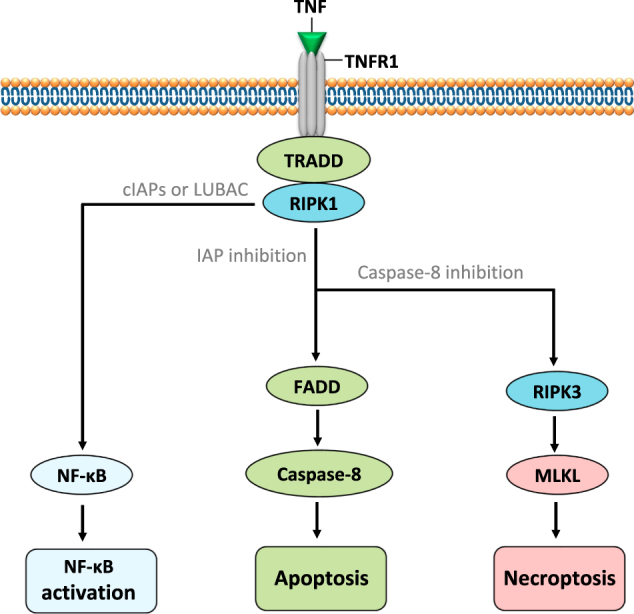
Necroptosis signaling pathway induced by TNFR1 stimulation via TNF TNF is a pleiotropic cytokine playing a key role in infection and tissue injury. TNF has a role not only in inflammation, since, under some conditions, as a consequence of TNFR1 ligation, it can also trigger regulated cell death pathways such as apoptosis or necroptosis

TNFR1 is one of the death receptors in the TNF superfamily and much of our knowledge of necroptosis comes from studies of the TNFR1 signaling pathway (Fig. 4)^1^. TNFR1 stimulation induces an early complex formation comprising TNFR1-associated death domain protein (TRADD) and RIPK1. Cellular inhibitors of apoptosis (cIAPs), including cIAP1 and cIAP2, and the linear ubiquitin chain assembly complex (LUBAC) are recruited to the initial complex, inducing Lys63-linked or linear ubiquitylation of RIPK1, respectively^2^. In consequence of ubiquitylation, the initial complex is stabilized, going on to activate Nuclear Factor-κB (NF-κB) transcriptional activity, which contributes to cell survival, proliferation, and the production of pro-inflammatory cytokines (Fig. 4)^1,2^. Conversely, inhibiting the ubiquitylation of RIPK1 through blocking of cIAPs (Smac mimetics or genetic deletion of IAPs)^37–40^ facilitates the formation of a different complex composed of TRADD, RIPK1, and oligomerized FAS-associated death domain protein (FADD)^2^. This second complex then recruits and activates caspase-8, finally inducing an RIPK1 activity-dependent apoptosis (Fig. 4). Moreover, LUBAC inhibition^2,41^, transforming growth factor-β-activated kinase 1 inhibition^42^, NF-κB essential modulator silencing^43^, or Pellino silencing^44^ also favor the formation of a FADD-containing complex and thus promote apoptosis^1^.

Following the stimulation of apoptosis, if caspase-8 activity is blocked, RIPK1 recruits RIPK3 through interactions between the RIP homotypic interaction motif (RHIM) domains. This RIPK3-containing complex is termed the necrosome. The RIPK3 can subsequently recruit and phosphorylate MLKL to trigger necroptosis (Fig. 4)^6,9^. MLKL, a downstream target of RIPK3, is considered as the effector of necroptosis^6,9^. RIPK3 can activate MLKL and induce its oligomerization, which facilitates oligomerized MLKL translocation to the plasma membrane and triggers cell permeabilization and necroptosis^45–48^.

Some additional proteins have been reported to be involved in the function of the necrosome. The FADD-like interleukin (IL)-1β-converting enzyme (FLICE)-inhibitory protein (FLIP) plays an important role in the control of necroptosis induced by TNF^2,38,49^, since caspase-8—FLIP heterodimers are believed to inhibit necroptosis by disrupting RIPK1-RIPK3 necrosome formation^2,49^. Cylindromatosis protein (CYLD) is an ubiquitin-editing enzyme which can remove ubiquitin chains from RIPK1 to facilitate the induction of necroptosis^50,51^. Upon TNF stimulation, CYLD can trigger necroptosis by promoting necrosome formation, phosphorylation and activation of RIPK3^51^, but caspase-8 can cleave CYLD to suppress necroptosis^50^. Moreover, heat shock protein 90 and its co-chaperone CDC37 complex are required for RIPK3 activation during necroptosis^52^. In contrast, both the phosphatase PPM1B^53^ and the ubiquitin-modifying enzyme A20^54^ can negatively regulate necroptosis through suppressing RIPK3 activity.

## TNF-induced necroptosis in neutrophils

A TNF-induced neutrophil necroptosis was reported recently which is dependent on a RIPK1- RIPK3-MLKL signaling^31^. In that work, the authors showed that in mouse neutrophils responding to high concentrations of TNF, X-linked IAP (XIAP) plays a crucial role in determining the type of neutrophil death, i.e. either apoptosis or necroptosis^31^. XIAP and cIAPs are all members of the IAP family. Like the cIAPs, XIAP was also reported to ubiquitylate RIPK1 and trigger NF-κB activation^2^. Thus, blocking XIAP could also facilitate the formation of the FADD-containing complex to induce either apoptosis or necroptosis in response to TNF (Fig. 4)^2,37^. In other cell types, however, evidence shows that the role of XIAP in restricting TNF-induced death is dispensable, because loss of XIAP fails to trigger TNF-induced apoptosis in mouse embryonic fibroblasts^39^ and cIAP1 inhibition alone is a sufficient induction for either apoptosis or necroptosis upon TNF stimulation^37,39^.

Moreover, in mouse neutrophils, the inhibition of XIAP results also in increased ubiquitylation of RIPK1 instead of the reduced ubiquitylation observed when cIAPs are blocked^31^. In line with these findings in mouse neutrophils, XIAP has also been reported to restrict TNF and RIPK3-dependent cell death in dendritic cells^55^ and to limit TLR and TNFR1-induced necroptosis in macrophages^56^. Similarly, loss of XIAP can also lead to elevated ubiquitylation of RIPK1^55^. Taken together, XIAP appears to be able to function independently of cIAPs in regulating the neutrophil death pathways and the complicated XIAP functions in different kinds of cells may well imply that the role of XIAP in regulating necroptosis is cell type dependent.

LPS can activate TLR4 to induce macrophage necroptosis when caspases are inhibited; here, autocrine TNF plays only a minor role^15^. However, in mouse neutrophils, it has been reported that only when cIAPs and XIAP are both blocked, can LPS trigger RIPK1-dependent apoptosis, which is then almost completely dependent on autocrine TNF^31^. Thus, XIAP can maintain neutrophil viability when cIAPs are blocked in response to LPS. RIPK1-dependent apoptosis initiated by LPS-induced autocrine TNF can then switch to an RIPK3 and MLKL-dependent necroptosis when caspases are inhibited^31^. However, these authors also show that low concentrations of TNF can also induce neutrophil cell death when cIAPs alone, or both cIAPs and XIAP, are blocked by Smac mimetics^31^. Accordingly, this cell death is probably an RIPK1-dependent apoptosis. Thus, it seems reasonable that low concentrations of TNF should also be able to induce neutrophil necroptosis in the presence of Smac mimetics when caspases are also inhibited. This, however, needs to be confirmed. Furthermore, we cannot exclude a role for XIAP in maintaining neutrophil viability by blocking LPS-induced TNF production when cIAPs are functionally absent. This hypothesis is also suggested by XIAP restriction of granulocyte/macrophage colony-stimulating factor (GM-CSF)-primed neutrophil death in response to TNF and increased XIAP expression upon GM-CSF stimulation^31^. Finally, the execution of necroptosis in mouse neutrophils appears to be dependent on MLKL translocation to the plasma membrane^31^. However, the role of ROS in TNF-induced necroptosis in mouse neutrophils has so far not been well investigated. Moreover, it should be noted, that TNF-induced necroptosis in human neutrophils has not been demonstrated so far.

## The mechanism of neutrophil necroptosis induced by adhesion receptors

Ligation of adhesion receptors, including CD44, CD11b, CD18, and CD15, can induce necroptosis in human neutrophils if the cells have been exposed to GM-CSF (Fig. 3)^19^. This process is characterized by cytoplasmic vacuolization, a phenomenon seen both in vitro and in vivo under inflammatory conditions^19,57^. This necroptosis pathway involves a RIPK3–MLKL—p38 MAPK–PI3K axis, for which all these molecular components are required to generate ROS via NADPH oxidase and subsequent necrosis. It is unknown how RIPK3 is activated by adhesion receptors. TLR stimulation can induce necroptosis by the TRIF-RIPK3 interaction through their RHIM domains (Fig. 2)^14,15^, for which TRIF may also play a role by activating RIPK3 in the neutrophil necroptosis induced by adhesion receptors. Clearly, this pathway needs confirmation. While the essential roles of p38 and PI3K in the activation of the NADPH oxidase within neutrophil cell death pathways had been reported earlier^57–59^, it remains unclear how the RIPK3-MLKL complex is able to bring about p38 MAPK activation.

Increased ROS production is indispensable for neutrophil necroptosis, because neutrophils from patients with chronic granulomatous disease (CGD) exhibiting a genetic defect in NADPH oxidase were unable to undergo necroptosis induced by adhesion receptors^19^. The strong increase in ROS production in human neutrophils may result in irreversible damage to biomolecules and is therefore severely deleterious for the cell. However, a direct MLKL-mediated plasma membrane disruption does not seem to occur. Instead, MLKL, perhaps together with RIPK3, was required to activate p38 MAPK, PI3K and NADPH oxidase, which finally triggered ROS production and neutrophil death. Therefore, MLKL should be seen not only as a necroptosis executor protein, but also as an adaptor protein for necrosis signaling.

In line with this idea, it has been shown that MLKL is able to activate p38 MAPK and NADPH oxidase, leading to regulated necrosis induced by adhesion in human eosinophils^60^. The execution of both neutrophil and eosinophil necroptosis appears to involve an ROS-dependent permeabilization of vacuole membranes, intracellular structures containing toxic granule proteins^20,57,60^. Moreover, MLKL can contribute to inflammasome activation^56^. In addition, RIPK3 fails to trigger phosphorylation on the mitochondrial protein phosphatase PGMA5S in the absence of MLKL^61^ and overexpression of MLKL was reported to activate JNK^9^. Interestingly, at the site of infection, wild-type mouse neutrophils are able to clear *S. aureus* while neutrophils lacking MLKL, like human neutrophils from CGD patients, kill microbes poorly^62,63^. Furthermore, this cell death pathway appears to be independent of autocrine TNF and can be induced without blocking IAP or caspase activities. Thus, the mechanism of necroptosis induced by adhesion receptors is quite different from TNF-induced necroptosis, though this cell death pathway does appear to be a close analogy to the necroptosis observed under physiological conditions. Clearly, the mechanism of RIPK3 activation by adhesion receptors in cytokine-primed neutrophils should be further investigated.

## The role of MSU crystals in neutrophil necroptosis

Neutrophil necroptosis has also been shown to be induced by phorbol 12-myristate 13-acetate (PMA) treatment or exposure to MSU crystals^32,33^. This cell death, measured 2 h after stimulation, is triggered by ROS production which further activates RIPK1, RIPK3, and MLKL. Thus, ROS are upstream of RIPK1-RIPK3-MLKL signaling and are believed to be initiators of the necroptosis induced by PMA or MSU crystals (Fig. 3). This cell death, whether induced by PMA or MSU crystals, can be inhibited by necrostatin-1 (a RIPK1 inhibitor) or necrosulfonamide (NSA; a MLKL inhibitor)^6,13^. However, these findings have been contradicted in a publication arguing that NSA was unable to block human neutrophil death in response to PMA under the same stimulation conditions^64^.

On the other hand, neutrophil necroptosis induced by MSU crystals was shown to be reduced by either necrostatin-1 treatment or by silencing the *Ripk3* gene in experimental mouse gouty arthritis models^32,65^. Furthermore, MSU crystals have also been shown to induce necroptosis in other cell types^66^. Clearly, more work needs to be done to confirm the role of ROS as initiators of neutrophil necroptosis (Fig. 3). This is also true, because it has also been shown that MLKL can be proximal to the generation of ROS in TNF-induced necroptosis^9^ and RIPK3 triggered ROS production plays an important role in myocardial necroptosis^67^.

NETs were first interpreted as a mechanism for fighting microbial infection^68,69^. Subsequently, it was found that NETs are generated in the course of neutrophil necroptosis induced by PMA or MSU crystals^32,33^. In contrast, another report described NET formation induced in live neutrophils independent of RIPK3 and MLKL^64^. One explanation may be that the so-called NET release during necroptosis is in fact a necrosis-related, passive release of chromatin^32,33^. So interpreted, NET formation would be independent of necroptosis^64^. In fact, it is unlikely that necroptosis would be beneficial for the host under conditions where NET formation would seem to be an appropriate anti-microbial response^70^.

## The mechanism of RIPK3-dependent regulated necrosis induced by phagocytosis of *S. aureus1* in neutrophils

Human neutrophils can phagocytose community-associated methicillin-resistant *S. aureus* (CA-MRSA) strain USA300 to fight infection. Some ingested *S. aureus* may survive within the neutrophils phagosome, however, preventing apoptosis and resulting in a necrosis-like cell death. Such human neutrophil death is considered to be an RIPK1-dependent necroptosis based on the inhibition of lysis by Nec-1^34^. However, later, the same authors found that cell lysis inhibited by Nec-1 is owing to its off-target effects and that this necrosis-like death in neutrophils partially requires RIPK3 activity, though independent of RIPK1 and MLKL^35^. These authors did not identify the executioner protein in this RIPK3-dependent necrosis and they considered this regulated cell death pathway to be distinct from necroptosis. However, ischemia- and oxidative stress-induced myocardial necroptosis is also independent of RIPK1 and MLKL, acting through the RIPK3-Ca^2+^-calmodulin–dependent protein kinase (CaMKII) pathway^67^. Thus, lysis of neutrophils by CA-MRSA may also be interpreted as one type of RIPK3-dependent necroptosis (Fig. 3). This RIPK3-dependent regulated necrosis induced by phagocytosis of *S. aureus* in human neutrophils is believed to facilitate the release of DAMPs and to allow the escape of viable bacteria leading to an amplification of local tissue damage and persisting infection^34^.

## The role of necroptosis in neutrophil-associated disorders

It has been predicted that necroptosis is involved in disease pathogenesis since studies in animal disease models provided strong evidence for this hypothesis. However, the role of necroptosis in human pathologies remains to be further identified^1,2^. Neutrophils are involved in many kinds of diseases; unlike apoptosis, necrosis of neutrophils can be very harmful during inflammation due to the liberation of their toxic contents and ROS production, which induce further tissue damage and an amplified inflammatory response. Furthermore, neutrophils undergoing necrosis may allow pathogens to escape from dead cells, inducing further infections^30^. Thus, an understanding of neutrophil necroptosis undoubtedly promises an approach for preventing neutrophil-associated excessive tissue injury or inflammatory disease by targeting key proteins in the necroptosis pathway.

The induction of necroptosis occurring under in vivo inflammatory conditions has been explored and the migration of human neutrophils to inflammatory sites was found to activate the RIPK3-MLKL pathway in tissue samples from patients with neutrophilic diseases including cutaneous vasculitis, ulcerative colitis, and psoriasis^19^. These diseases are characterized not only by neutrophilic tissues, but also by strong triggers of inflammation, which involve autoimmune mechanisms^71–73^. Although tissue samples from patients with neutrophilic diseases are without any experimental stimulation, adhesion receptors^19,57^, including CD44, CD11b, CD18, CD15, and Siglec-9 may be the triggers for neutrophil necroptosis, because the cytoplasmic vacuolization of neutrophils can also be observed in inflamed tissue samples of these patients^57^. Moreover, it has been previously demonstrated that hyaluronan, a natural ligand of CD44, is able to trigger the necroptotic pathway in neutrophils^57^. However, exactly when necroptosis triggering occurs is not clear, since it could be during migration, but could also be later at the site of inflammation. Thus, adhesion receptors, RIPK3 and MLKL are all potential therapy targets.

Neutrophil necroptosis seems also to be involved in the pathology of gout, because, in the mouse, gouty arthritis models based on the injection of MSU crystals into subcutaneous air pouches exhibit the gout-like tophus formation induced by neutrophil necrosis which can be reduced both by necrostatin-1 treatment or by silencing of the *Ripk3* gene^32^. Thus, RIPK1, RIPK3, and MLKL might serve as molecular targets for gout therapy. Furthermore, in patients diagnosed with X-linked lymphoproliferative syndrome type 2 (deficient in XIAP), neutrophil necroptosis may play a role in disease progression^31^, and TNF, XIAP, RIPK1, RIPK3, and MLKL promise to be therapy targets. However, the role of TNF, adhesion receptors, MSU crystals, and phagocytosis-induced neutrophil necroptosis in human diseases remains to be further investigated.

## Conclusions

The identification of the necroptosis death pathway helps us to better understand overall necrotic cell death, which is not always an accidental or uncontrolled process, and which appears to play an important role in inflammation and disease pathogenesis. The characterization of the neutrophil necroptosis signaling pathway may facilitate a better control of tissue damage or excessive inflammation induced by neutrophil dysfunction, helping us to identify appropriate drug targets in neutrophil-associated disorders such as cutaneous vasculitis, ulcerative colitis, and psoriasis. Although necroptosis has been extensively studied, neutrophil necroptosis has so far been little investigated. Given the important role of neutrophils in the immune system and in different pathologies, it is worthwhile to explore the mechanisms, as well as the triggers and key proteins of neutrophil necroptosis. A better understanding of this signaling pathway will hopefully benefit the treatment of inflammatory or autoimmune diseases in the future.
